# Pressure-Dependent Pneumothorax Following Pleural Drainage in a Non-expandable Lung: A Case Report and Literature Review

**DOI:** 10.7759/cureus.89587

**Published:** 2025-08-07

**Authors:** Ahmed R Fadel, Seshnag Siddavaram, Sudhir Lohani

**Affiliations:** 1 Respiratory Medicine, Dartford and Gravesham NHS Trust, Dartford, GBR; 2 Acute Internal Medicine, Dartford and Gravesham NHS Trust, Dartford, GBR

**Keywords:** air leak management, chest tube management, lung adenocarcinoma, non-expandable lung, pleural elastance, pleural manometry, pneumothorax ex vacuo, pressure-dependent pneumothorax, thoracentesis complication, trapped lung

## Abstract

Pressure-dependent pneumothorax is an under-recognized but clinically significant phenomenon that complicates pleural fluid drainage, particularly in patients with non-expandable lungs due to malignancy or chronic pleural fibrosis. Unlike pressure-independent pneumothorax, this condition arises from the pronounced transpleural pressure gradient generated during therapeutic thoracentesis or chest drainage. This negative pressure transiently distorts the visceral pleura, allowing air to enter the pleural space until an equilibrium is reached. The resulting pneumothorax typically remains stable and resolves spontaneously without intervention. However, it is frequently misdiagnosed as a pressure-independent pneumothorax, leading to unnecessary interventions, such as chest drain insertion or even surgical management.

We report the case of a 65-year-old man with stage IV lung adenocarcinoma and malignant pleural effusion who developed basilar pneumothorax after chest drainage. Although the air leak was initially presumed to be iatrogenic and was treated with negative suction, persistent bubbling and minimal radiographic changes prompted re-evaluation. Clamping of the chest drain and serial imaging confirmed stable pneumothorax with fluid reaccumulation, consistent with pressure-dependent physiology. Removal of the drain resulted in no progression. This case highlights the importance of recognizing pressure-dependent pneumothorax to avoid unnecessary intervention. Awareness of this mechanism, together with pleural manometry or clinical criteria, can inform individualized management, prevent prolonged air leaks, and improve outcomes in patients undergoing pleural drainage.

## Introduction

Pneumothorax remains one of the most common complications of pleural interventions, with an incidence approaching 20% following therapeutic thoracentesis and even higher rates after endobronchial valve placement or lung resection. Historically, most postprocedural pneumothoraces have been attributed to persistent structural defects such as bronchopleural or alveolopleural fistulas. A bronchopleural fistula (BPF) is a pathological communication between the main stem, lobar, or segmental bronchus and the pleural space, which is typically associated with significant morbidity and often requires surgical intervention. In contrast, an alveolopleural fistula (APF) represents a communication between the distal pulmonary parenchyma beyond the segmental bronchus and pleural space and usually resolves without operative management [1].

Pressure-dependent pneumothorax is a distinct and under-recognized mechanism in which excessive negative intrapleural pressure arises due to a mismatch between lung and chest cavity volumes, often following pleural fluid drainage or surgical procedures. This negative pressure transiently distorts the visceral pleura, resulting in self-limiting APFs. These air leaks typically resolve spontaneously as the pressure equilibrates, rendering continued suction unnecessary and potentially harmful. Misdiagnosis of a pressure-independent pneumothorax can lead to avoidable morbidity, including prolonged hospitalization, patient discomfort, and unnecessary intervention [2,3]. Here, we describe a representative case of right-sided pressure-dependent pneumothorax following pleural drainage in a patient with a non-expandable lung and highlight diagnostic strategies that integrate pleural manometry with clinical criteria to distinguish benign, pressure-dependent air leaks from persistent fistulas requiring intervention.

## Case presentation

A 65-year-old man, an active smoker with a 30-pack-year history, presented with progressive shortness of breath. On examination, the patient was hemodynamically stable but required 2 L of oxygen via a nasal cannula to maintain adequate saturation. Chest auscultation revealed markedly decreased entry of air into the right hemithorax. Percussion of the right hemithorax revealed a dull stony note, consistent with a large pleural effusion.

Two weeks prior to admission, he underwent therapeutic thoracentesis for a large symptomatic right-sided pleural effusion, during which 1.5 liters of straw-colored fluid were drained. Pleural fluid analysis revealed exudative pleural effusion (Table 1).

**Table 1:** Pleural fluid biochemical analysis, reference ranges, and interpretation. The elevated LDH (lactate dehydrogenase) and protein levels meet Light’s criteria, confirming the presence of an exudative pleural effusion.

Parameter	Result	Reference Range	Comment	Interpretation
LDH	618 U/L	<200 U/L (typically)	Elevated	Exudative effusion (LDH > 2/3 upper limit of serum)
Protein	46 g/L	<30 g/L (transudate cutoff)	Elevated	Exudative effusion (protein >30 g/L)

Cytological evaluation revealed numerous atypical cells with enlarged pleomorphic nuclei, prominent nucleoli, and abundant foamy eosinophilic cytoplasms. Immunohistochemistry revealed that the tumor cells were positive for cytokeratin 7 (CK7), Ber-EP4, epithelial membrane antigen (EMA), and thyroid transcription factor-1 (TTF-1). CK7 is expressed in many epithelial-derived tumors, including those originating in the lungs. Ber-EP4 is an antibody that targets the epithelial cell adhesion molecule (EpCAM), which is found in most epithelial cells and carcinomas. It is particularly useful for differentiating basal cell carcinoma, which is typically Ber-EP4 positive, from squamous cell carcinoma, which is usually Ber-EP4 negative. EMA is a marker of epithelial differentiation that supports the diagnosis of carcinoma. TTF-1 is a nuclear marker expressed in lung and thyroid epithelial cells and is highly specific for lung adenocarcinoma. Collectively, these findings support the diagnosis of metastatic adenocarcinoma of the primary lung origin.

Staging computed tomography (CT) and histopathological assessment confirmed the diagnosis of stage IV lung adenocarcinoma (T3N2M1a) with malignant right-sided pleural effusion. Additional molecular profiling identified a BRAF V600E mutation with no detectable alterations in EGFR, ALK, ROS1, or MET and demonstrated high PD-L1 expression (>50%), which is predictive of a favorable response to immune checkpoint inhibitors such as pembrolizumab.

At the time of presentation, the patient denied experiencing chest pain, fever, or hemoptysis. Laboratory investigations revealed elevated inflammatory markers, with a C-reactive protein (CRP) level of 122 mg/L; however, procalcitonin was negative. The blood-stained pleural fluid showed no microbial growth in culture. Sputum and blood cultures were negative. Notably, the CRP level showed a transient rise following the pleural procedure but gradually declined after fluid drainage without the initiation of antibiotic therapy. This pattern suggests that the elevated CRP level was likely attributable to the malignant pleural effusion itself rather than an underlying infectious process (Table 2).

**Table 2:** Summary of key blood test results. Routine blood investigations showing negative procalcitonin, improving CRP following drainage of malignant pleural effusion without antibiotic treatment, and stable chronic kidney disease parameters.

Test	December 12, 2024	December 15, 2024	December 17, 2024	December 19, 2024	December 20, 2024	December 23, 2024	December 27, 2024	Reference Range
Creatinine (µmol/L)	140	141	117	133	122	122	128	60–110 µmol/L
WBC (x10^9/L)	7.2	7.4	7.1	6.3	6.1	6.2	7 3	4.0–11.0 ×10^9/L
Neutrophils (x10^9/L)	5.3	5.1	5.6	4.8	4.4	4.5	5.1	2.0–7.5 ×10^9/L
CRP (mg/L)	122	113	166	121	93	44	31	<5 mg/L
Procalcitonin (ng/mL)	–	–	0.09	–	–	–	–	<0.1 ng/mL

**Figure 1:**
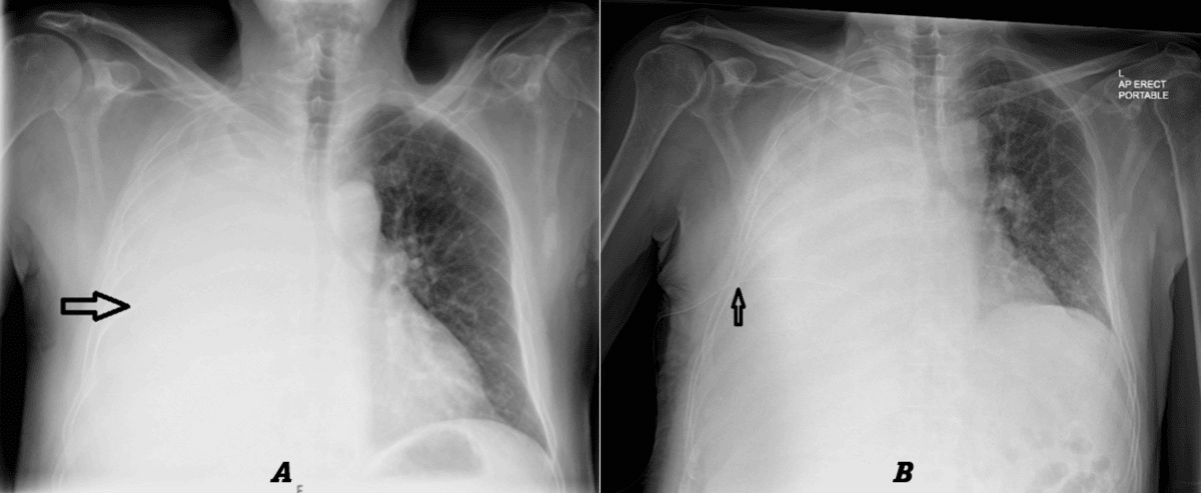
Chest X-ray: before and after chest drain insertion. A: Chest X-ray: Complete white-out of the right hemithorax. B: Chest X-ray performed after chest drain insertion confirmed the drain was in situ (arrow) and demonstrated no pneumothorax.

Chest radiography performed on admission revealed complete whitening of the right hemithorax (Figure 1A). A chest drain was inserted, and approximately 1.5 liters of straw-colored fluid were drained before clamping the drain. After the removal of approximately one liter, the patient began experiencing chest discomfort. The chest drain was swinging but not bubbling. Chest radiography performed after drain insertion confirmed that the drain was in situ and showed no pneumothorax (Figure 1B).

**Figure 2:**
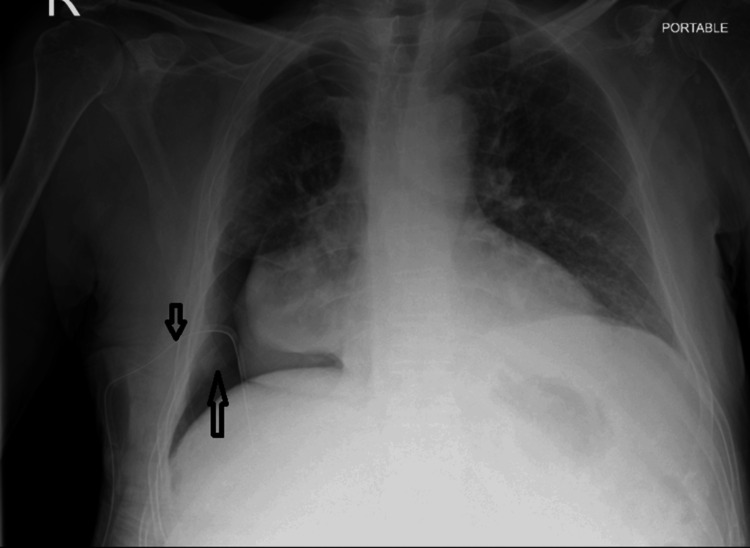
Chest X-ray demonstrated the chest drain in situ with a right-sided basilar pneumothorax.

Two days after chest drain insertion, the underwater seal demonstrated minimal bubbling, which was observed primarily during forceful coughing. A repeat chest radiograph revealed right-sided basilar pneumothorax (Figure 2).

**Figure 3:**
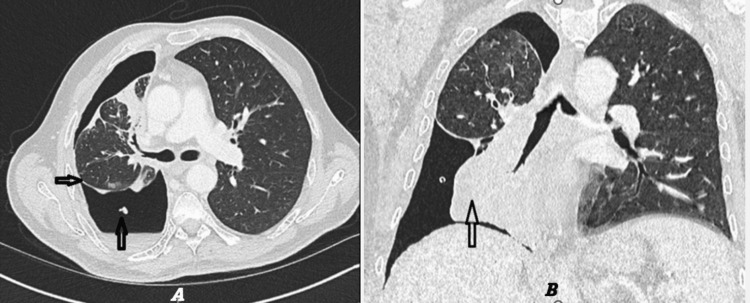
CT chest after chest drain insertion. A: CT chest (axial view) demonstrated a right hydropneumothorax with associated pleural thickening and the chest drain in situ, and ipsilateral (right sided) mediastinal shift. B: CT chest (coronal view) demonstrated a perihilar mass resulting in complete collapse of the right middle and lower lobes.

Subsequently, contrast-enhanced CT of the thorax was performed for further assessment. The scan demonstrated a right hydropneumothorax with further collapse and consolidation of the remaining right lung parenchyma. There was a marked mediastinal shift toward the right hemithorax, and the chest drain remained in situ. Additionally, a right hilar mass could not be excluded as a contributing factor to persistent airway compression and volume loss (Figures 3a, 3b).

Because bubbling was evident in the drain during forceful coughing, pneumothorax was initially presumed to be iatrogenic, and the drain was connected to negative suction.

**Figure 4:**
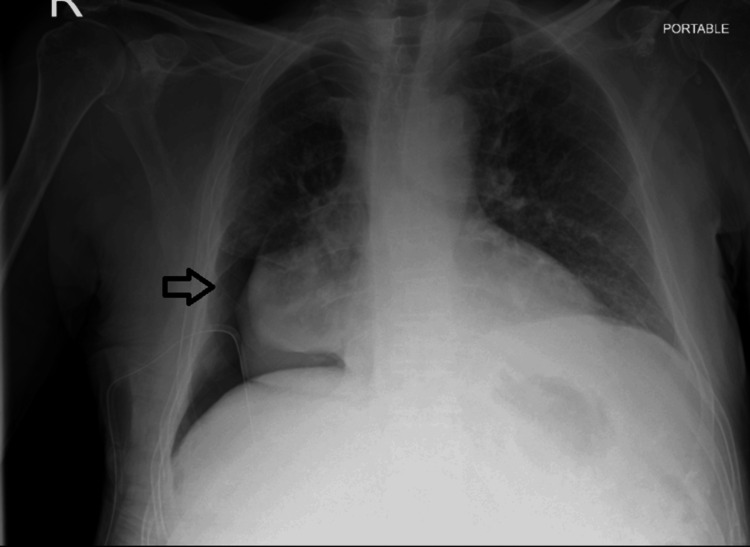
Chest X-ray performed two days after initiation of suction demonstrated a slight reduction in the size of the pneumothorax.

The patient remained under suction for two days. During this period, continuous bubbling of the chest drain persisted, and the patient continued to report persistent chest pain. A follow-up chest X-ray two days after suction demonstrated only a slight reduction in the size of the basilar pneumothorax (Figure 4).

**Figure 5:**
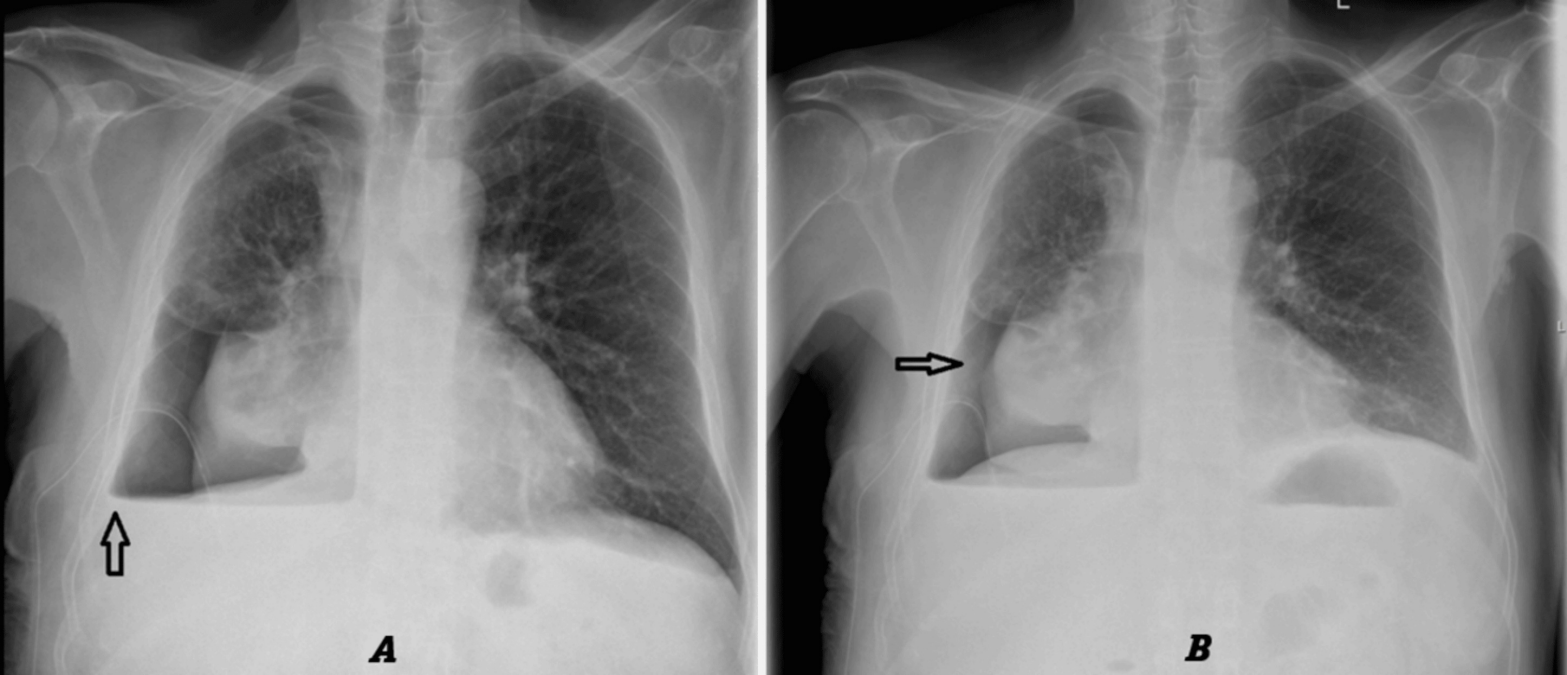
Chest X-ray after 24 hours off suction and immediately following clamping of the chest drain and four hours later. A: Reaccumulation of pleural effusion, consistent with equilibration of intrapleural pressure. B: No increase in the size of the hydropneumothorax.

Following reassessment, a pressure-dependent pneumothorax was suspected, and it was recognized that applying suction in this context was inappropriate. As pleural manometry was unavailable, clinical criteria for pressure-dependent pneumothorax were used to guide management. The chest drain was clamped to support the diagnosis, and chest radiographs were obtained immediately after clamping (Figure 5a) and again four hours later (Figure 5b). These images showed no interval change in the size of the pneumothorax but did reveal reaccumulation of the right-sided pleural effusion. During this period, the patient remained clinically stable and reported an improvement in chest pain following chest drain clamping.

**Figure 6:**
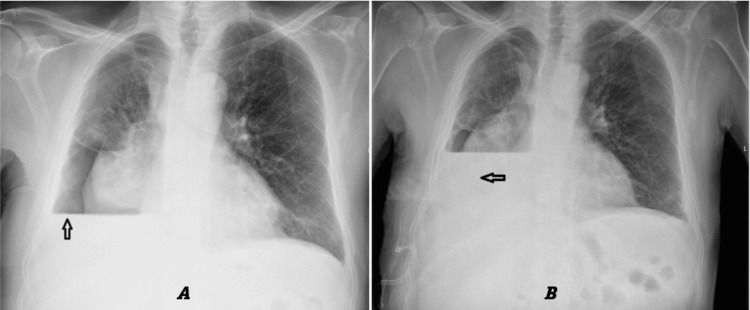
A chest X-ray was performed after removal of the chest drain and after insertion of the indwelling pleural catheter (arrow). A: Chest X-ray performed 24 hours after removal of the chest drain demonstrated an increase in the size of the pleural effusion. B: Chest X-ray following insertion of an indwelling pleural catheter.

After unclamping the drain, the patient was asked to cough forcefully. No bubbling was observed in the underwater seal, suggesting equilibration of pleural pressure and cessation of air leaks. Subsequently, the chest drain is removed. Chest radiography performed the following day demonstrated no progression of pneumothorax and showed an increase in pleural effusion volume (Figure 6a).

Four days later, an indwelling pleural catheter was inserted (Figure 6b), and the patient commenced scheduled drainage of the effusion three times per week, coordinated by the district nursing team. He was subsequently followed up by the oncology team and started on systemic anticancer therapy.

## Discussion

Pressure-dependent pneumothorax occurs when pleural fluid drainage creates a mismatch between the lung volume and thoracic cavity size, resulting in localized lung deformation. This deformation transiently opens alveolopleural connections, allowing air to leak into the pleural space until pressures equilibrate. The air leak then stops spontaneously once the deformation resolves. This process, sometimes termed pneumothorax ex vacuo, typically remains stable and often demonstrates fluid reaccumulation rather than progressive air accumulation [2].

In contrast, pressure-independent pneumothorax is caused by a persistent bronchopleural or alveolopleural fistula acting as a ball-valve mechanism. This permits unidirectional air entry into the pleural cavity, leading to progressive accumulation of air, development of positive intrapleural pressures, and risk of tension pneumothorax or complete lung collapse. Unlike pressure-dependent cases, these air leaks do not self-limit and require active intervention, such as continuous drainage or surgical repair [2,3].

Pathophysiology of pressure-dependent pneumothorax

Pressure-dependent pneumothorax arises during pleural fluid drainage when a pronounced mismatch between the lung size and thoracic cavity prevents the lung from fully expanding to contact the chest wall. This mismatch creates a substantial parenchymal-pleural pressure gradient, distorting the subpleural alveolar structures and transiently opening parenchymal-pleural fistulas (PPFs). As fluid removal further lowers the intrapleural pressure, air enters the pleural space through these fistulas, resulting in pneumothorax [2].

In contrast to pressure-independent pneumothorax, where a persistent unidirectional air leak occurs due to a bronchopleural or alveolopleural fistula, air leaks in pressure-dependent pneumothorax are brief and resolve once the pressure gradient normalizes or suction is discontinued [4,5].

Pleural manometry and pressure-volume curves clearly illustrate this mechanism. Pressure-volume tracings of a non-expandable lung demonstrated a biphasic pattern: an initial gradual pressure decline followed by an abrupt drop as lung deformation-triggered air entry. Continuous manometry further shows that coughing or additional drainage can transiently increase the intrapleural driving pressure (transpulmonary pressure), producing short-lived air leaks that resolve once the pressures equilibrate [2,5].

This pattern indicates that the visceral pleura remains structurally intact and that pneumothorax in this setting results purely from physiological pressure gradients rather than true pleural injury [2]. Recognizing this benign mechanism is essential, as applying negative suction can unnecessarily prolong air leaks and extend hospitalization.

Diagnosis of pressure-dependent pneumothorax

Pressure-dependent pneumothorax can be diagnosed either by pleural manometry or by applying clinical criteria alone.

Pleural Manometry

When available, pleural manometry, a tool that allows direct measurement of pressure within the pleural space in the presence of pleural effusion or pneumothorax, provides valuable physiological confirmation. Continuous pleural pressure tracings can be obtained by connecting a pressure transducer or digital manometer to the chest drain while the drain is clamped. Clamping is essential because leaving the system open to suction or under a water seal prevents accurate assessment and hinders the ability to distinguish between pressure-dependent and pressure-independent pneumothorax. Moreover, manometry cannot be reliably interpreted if the chest drain system has a leak or if air tracks around the chest tube into subcutaneous tissue, as these introduce spurious pressure changes and invalidate tracing [2,6].

Clinical Criteria

The characteristic findings of a pressure-dependent air leak include stable end-expiratory plateau pressure after drainage or clamping without progressive rise (unlike pressure-independent leaks, which show continuous increases), transient increases in pleural pressure during coughing, and resolution of the air leak once suction is stopped and pressure equilibrates. This reproducible pattern reassures clinicians that structural pleural defects are also present. If manometry is unavailable, a diagnosis can be established using the combined clinical and radiographic features. Radiographic criteria included pneumothorax localized to the side of the procedure, absence of contralateral mediastinal shift, and stable pneumothorax size on serial chest radiographs performed at least four hours apart after drainage stops or when the drain was clamped.

Clinically, there should be a known risk factor (such as trapped lung, lobar collapse, or prior partial lung resection), absence of symptoms typically associated with pneumothorax (dyspnea or hemodynamic compromise), chest radiographic findings as described, and no continuous air leakage when suction is not applied. An evidence-based diagnostic algorithm guides management: if the patient is unstable, pneumothorax is managed conventionally. If stable, manometry demonstrating stable plateau pressures with cough-related dips would support a pressure-dependent leak. When manometry data were unavailable, a clinical criteria checklist was applied [2].

Management

If the criteria were met, suction was avoided, and the drain could be removed if no symptoms persisted; however, if the criteria were not met or the diagnosis remained uncertain, the air leak was classified as conventional. This approach, as described in an interesting study, emphasizes observation over intervention to avoid unnecessary procedures and to reduce the risk of prolonged hospitalization [2].

Pressure-dependent pneumothorax occurs in clinical scenarios characterized by impaired lung re-expansion and a significant parenchymal-pleural pressure gradient. Common settings include thoracentesis in non-expandable lungs, partial lung resections (e.g., lobectomy or segmentectomy), and bronchoscopic lung volume reduction (BLVR) with endobronchial valves in severe emphysema. In these cases, factors such as pleural restriction, loss of elastic recoil, or regional air trapping create a size mismatch between the lung and the thoracic cavity during drainage. This mismatch can distort the lung periphery and transiently form PPFs, leading to physiologically driven air leaks rather than structurally permanent ones [2,7].

Pressure-dependent pneumothorax secondary to thoracentesis in patients with non-expandable lungs

Unlike iatrogenic pneumothorax caused by direct pleural puncture or persistent APF, pressure-dependent pneumothorax arises when there is a mismatch between the size of the lung and the thoracic cavity during fluid evacuation. As large volumes of pleural effusion are removed, the intrapleural pressure becomes markedly negative, exerting traction on the visceral pleura. This deformation transiently opens pre-existing microfistulas or distends the subpleural alveolar units, allowing air to enter the pleural space. Once drainage stops and the pressure normalizes, the air leak typically resolves without further intervention.

Clinically, this phenomenon is characteristically radiographically stable, often presents as basilar pneumothorax, and frequently demonstrates rapid reaccumulation of fluid if left untreated. Pleural manometry has been instrumental in clarifying this physiology; affected patients exhibit high pleural elastance (>14.5 cm H₂O/L), consistent with non-expandable lung behavior. Observational studies have confirmed that neither pre- nor post-thoracentesis chest radiographs reliably predict pleural elastance or the risk of pressure-dependent pneumothorax, underscoring the value of manometry when feasible.

Most patients are asymptomatic or experience only mild discomfort, and their condition is self-limiting. However, the misinterpretation of iatrogenic pneumothorax frequently leads to unnecessary chest tube insertion and suction, which can perpetuate air leaks and increase morbidity. A critical learning point is that the absence of progressive dyspnea, hemodynamic instability, or radiographic enlargement on serial imaging strongly supports a benign clinical course. Recognition of this mechanism is especially important when managing patients with trapped lungs or chronic pleural inflammation, as these conditions predispose patients to non-expandability. Awareness of pressure-dependent pneumothorax can help avoid inappropriate interventions, reduce the length of hospital stay, and improve patient safety [3,5,8,9].

Unexpandable Lung: Trapped Lung and Lung Entrapment

An unexpandable lung refers to the failure of the lung to re-expand after fluid drainage and includes both trapped lung and lung entrapment. A trapped lung develops after resolved pleural inflammation (e.g., prior empyema or hemothorax) leaves a fibrous visceral pleural peel restricting expansion. In contrast, lung entrapment results from active pleural diseases such as malignancy or infection. Differentiating between these entities is critical because trapped lungs do not benefit from pleurodesis, whereas entrapment may improve if the underlying process resolves.

Fibrosis reduces compliance in a trapped lung, and when pleural effusion accumulates, the lung remains compressed. During thoracentesis, fluid removal creates marked negative intrapleural pressure because the lung cannot expand to fill the space. This can lead to cough, pain, or pneumothorax due to transient alveolar-pleural fistula.

Pleural manometry remains the reference standard for assessing pleural elastance and for confirming an unexpandable lung. High elastance (>14.5 cm H₂O/L) and early steep pressure drops during drainage were characteristic. Recognizing this pattern guides safe drainage and informs the prognosis, particularly when planning pleurodesis or catheter placement. When manometry is unavailable, ultrasound can help to anticipate a trapped lung. B-mode imaging may show reduced lung sliding and diaphragmatic movement, whereas M-mode ultrasound identifies the absent sinusoid sign, indicating a lack of lung movement with respiration and predicting non-expandability with high specificity. Two-dimensional shear wave elastography has also been explored to measure pleural stiffness but remains investigational. Although ultrasound aids in prediction, no single sign reliably distinguishes trapped lungs from entrapment. Combining ultrasound findings with manometry and clinical assessment offers the most accurate diagnosis and guides management decisions [10-13].

Pressure-dependent pneumothorax after partial lung resection

Pressure-dependent pneumothorax is an under-recognized phenomenon that can occur after partial lung resection, such as lobectomy or segmentectomy. In a landmark case, Chopra et al. [14] demonstrated that, following lobectomy, what appeared to be a persistent air leak resolved spontaneously without progressive pneumothorax when the chest tube was clamped, confirming its pressure-dependent nature. Pleural manometry in this context was pivotal; with the drain on the water seal, cough-induced pressure dips produced transient air egress, followed by stabilization of pressures as equilibration occurred, indicating no persistent fistula requiring continuous drainage [14].

This insight builds on earlier work by Bronstein et al. [15], who showed that conservative management strategies, such as maintaining the chest drain on the water seal rather than applying suction, significantly reduced the hospital stay in patients with prolonged postoperative air leaks. In their study, patients managed without suction achieved faster chest tube removal and had a lower incidence of prolonged air leaks. These findings suggest that not every postoperative air leak reflects an active, pressure-independent fistula that requires intervention [15].

Accurate differentiation between pressure-dependent and pressure-independent pneumothorax, either by clinical criteria or, more definitively, by pleural manometry, has important implications. If pressure-dependent physiology is confirmed, suction should be avoided, and chest drains can often be removed safely and earlier, preventing unnecessary prolongation of drainage and hospitalization [2,4,14-18].

Pressure-dependent pneumothorax after endobronchial valve placement

BLVR using endobronchial valves has become an increasingly common intervention in patients with severe emphysema. Pneumothorax is one of the most frequent complications, occurring in up to one-third of the cases [2]. Traditionally, these pneumothoraces were assumed to result from alveolar rupture and persistent air leaks, analogous to pressure-independent pneumothorax. However, recent evidence demonstrates that pressure-dependent pneumothorax can also occur after BLVR, and distinguishing between these two mechanisms has important clinical implications [19].

In a prospective series by Chung et al. (2024) [19], approximately 50% of pneumothoraces following BLVR were found to be pressure dependent. Pleural manometry played a critical role in differentiating these cases: pressure-dependent leaks exhibited a stable plateau of end-expiratory pleural pressure with transient drops during coughing or additional drainage, and no progressive rise over time. In contrast, pressure-independent leaks showed continuously increasing end-expiratory pleural pressures, consistent with the ongoing unidirectional air entry through a persistent fistula.

Importantly, this distinction translates into meaningful differences in management and outcomes. Patients with pressure-dependent pneumothorax had shorter chest tube durations (6.6 vs. 23.4 days), shorter hospital stays (8.8 vs. 19.8 days), and required fewer repeat bronchoscopic interventions compared to those with pressure-independent leaks. These findings emphasize that pressure-dependent pneumothorax after valve placement is frequently self-limiting and does not require prolonged drainage or valve removal [2,19,20].

While the pressure-dependent pneumothorax mechanism has been classically described after procedures such as thoracentesis for pleural effusions associated with non-expandable lung and following lung resection, recent publications, including the review by Walker et al., highlight that similar physiological principles may apply to other pneumothorax types. In this context, the pressure gradient between the pleural space and alveoli, not merely structural defects, can be a major determinant of persistent air leaks.

This perspective aligns with the hypothesis by Walker et al. [21] that inserting a chest tube in a clinically stable pneumothorax patient can worsen the transpleural pressure gradient. Negative intrapleural pressure may perpetuate air leakage from small peripheral fistulas that may otherwise seal spontaneously if left undisturbed. This concept provides a physiological explanation for findings from randomized trials demonstrating that conservative management, observation without drainage, leads to faster resolution, fewer prolonged air leaks, and shorter hospital stays compared to routine tube thoracostomy [21].

In other words, eliminating the driving pressure gradient by avoiding drainage may promote the spontaneous healing of alveolar defects. This evolving understanding has likely contributed to the paradigm shift reflected in the most recent pneumothorax management guidelines from the British Thoracic Society (BTS) and the European Respiratory Society (ERS), both of which now recommend conservative management as the first-line approach for asymptomatic or minimally symptomatic primary spontaneous pneumothorax, regardless of its size [7,17,21,22].

Therefore, the pressure-dependent air-leak framework provides a unifying physiological explanation across various pneumothorax etiologies: procedural, postoperative, and spontaneous. Recognizing this shared mechanism highlights the need for individualized management strategies, supporting observation or water seal drainage in stable patients while reserving suction or invasive intervention for those with worsening symptoms or clinical deterioration.

## Conclusions

This case reinforces that applying negative suction in pressure-dependent pneumothorax is often counterproductive as it sustains the transpleural pressure gradient and prolongs the air leak. Conservative management, avoiding suction and considering early chest drain removal, can be both safe and effective. Importantly, the physiological principles outlined here extend beyond thoracentesis and may explain persistent air leaks following lung resection or endobronchial valve placement. Incorporating pleural pressure dynamics into clinical assessment enables more individualized care, reduces unnecessary interventions, and may shorten hospital stay. Clinicians should maintain a high index of suspicion for pressure-dependent pneumothorax to optimize outcomes and align management with emerging evidence and recommendations.
